# Socioeconomic inequality of low sexual autonomy among reproductive-age women in four selected sub-Saharan African countries: a decomposition analysis by using performance monitoring for action data

**DOI:** 10.3389/fgwh.2025.1719041

**Published:** 2026-01-16

**Authors:** Dessie Abebaw Angaw, Tigist Kifle Tsegaw, Nimrod Muhumuza, Gertrude Nakanwagi, Moses Mulumba

**Affiliations:** 1 Evidence Generation Department, Afya na Haki Institute, Kampala, Uganda; 2 Department of Epidemiology and Biostatistics, Institute of Public Health, College of Medicine and Health Sciences, University of Gondar, Gondar, Ethiopia; 3 Deputy Director General, Afya na Haki Institute, Kampala, Uganda; 4 Afya na Haki Institute, Kampala, Uganda

**Keywords:** Africa, equity, reproductive-age women, sexual autonomy, socioeconomic inequity

## Abstract

**Background:**

Sexual health is a vital component of overall well-being and life happiness. The ability of women to make independent decisions regarding consensual sexual relationships is essential for their empowerment and the achievement of reproductive rights. Globally, only 55% of women can make their own decisions about sexual and reproductive health (SRH). Socioeconomic factors such as age, income, education, and early marriage significantly influence sexual autonomy. Therefore, this study aims to assess socioeconomic inequalities in sexual autonomy among women of reproductive age in four sub-Saharan African countries, using recent Performance Monitoring for Action (PMA) data.

**Methods:**

This study analyzed data from four sub-Saharan African countries—Burkina Faso, Ethiopia, Kenya, and Uganda—using the PMA project dataset. A weighted sample of 17,855 women of reproductive age was included. The dependent variable was sexual autonomy, defined as the presence of choice in sexual decision-making. Socioeconomic inequality was measured using the concentration curve and concentration index. Additionally, decomposition analysis was conducted to determine the contribution of explanatory variables to the overall inequality.

**Results:**

The weighted Erreygers normalized concentration index for low sexual autonomy was calculated as −0.184, with a standard error of 0.021 (*P* < 0.0001). Similarly, the corresponding concentration curve lies above the line of equality, showing that sexual autonomy is disproportionately distributed among the poor. Decomposition analysis revealed that rural residence (38.62%), followed by media access (16.52%), lower wealth quintile (13.71%), women's education (8.87%), and husband's education (7.24%) contribute to the overall inequality.

**Conclusion:**

Socioeconomic inequality was evident in low sexual autonomy across the four countries. According to the decomposition analysis of this inequality, the primary contributor was rural residence, followed by media access, wealth quintile, women's education, and husband's education.

## 1 Introduction

Sexual health is an important part of a person's overall health and happiness. It encompasses the freedom to choose one's sexual partners and engage in consensual sexual activity (1). In this context, sexual autonomy refers to the fundamental human right to make informed and independent decisions about one's body, sexuality, sexual experiences, and fertility, free from coercion, discrimination, and violence (2, 3). Sexual autonomy signifies the existence of choice—the right or opportunity to make decisions about one's sexual life—while the exercise of choice pertains to the actual ability to act on those decisions, such as negotiating consent, refusing unwanted sex, or initiating sexual activity (4). It serves as a key indicator of women's empowerment, as a woman who can make decisions about her own body is more likely to have greater agency and influence in other areas of her life (5, 6).

The empowerment of women and girls, along with the full realization of their reproductive rights—an indicator of the Sustainable Development Goals (5.6.1)—depends on their ability to make autonomous decisions regarding consensual sexual relationships, contraceptive use, and access to sexual and reproductive health services (7). Having authority over one's own body is a fundamental human right. Women who lack autonomy over their sexual and reproductive health are more likely to experience intimate partner abuse and sexually transmitted diseases (STDs), and they frequently face challenges when seeking and using necessary medical care (8–10).

According to the United Nations Population Fund (UNFPA) report, globally, only 55% of girls and women are able to make their own decisions regarding sexual and reproductive health (SRH) (11). In some developed countries, the exercise of sexual choice exceeds 90% (12). A study from Sub-Saharan Africa found that 45.5% of women demonstrated sexual autonomy, with significant regional variation (13), while other studies reported rates of approximately 83.3% (14) and 73% (15). The proportion of women able to refuse sex ranged from 18.3% to 92.4%, and the percentage of women who could make at least one reproductive health decision—such as refusing sex, using contraception, or seeking healthcare—varied from 25.5% to 97.2% (16). Previous research has shown that sociodemographic and economic factors, including education, age, wealth status, and age at first marriage, as well as community, national health system, and interpersonal factors, are strongly correlated with the capacity to make autonomous decisions regarding consensual sexual relationships (17–21).

Comprehensive Sexuality Education (CSE) programs have been developed for teenagers aged 15–24 to protect sexual and reproductive health rights (SRHR) and promote informed decision-making (22). By 2030, Sustainable Development Goal (SDG) 5 aims to empower all women and girls and achieve gender equality. However, progress remains slow, with only 15.4% of the country currently on track and 23.1% significantly behind the target. At this rate, achieving full gender equality will take longer than anticipated (23). Indicator 5.6.1 of the SDGs, which measures women's autonomy in making informed decisions about sexual relations, contraceptive use, and access to reproductive health care, remains very low in sub-Saharan Africa, with average coverage around 38% in 2024, compared to over 80% in some European, Latin American, and Caribbean countries (24). Therefore, this study aims to assess socioeconomic inequalities in sexual autonomy among reproductive-age women in four sub-Saharan African countries, using the most recent phase of PMA data. The findings will assist public health planners and policymakers in addressing socioeconomic disparities and designing targeted interventions for different socioeconomic groups. This approach will help empower women and promote gender equality.

## 2 Methods and materials

### 2.1 Data source

This study utilized data from the Performance Monitoring for Action (PMA) project, which collects data at both household and health facility levels across nine African and two Asian countries participating in the Family Planning 2020 (FP2020) initiative. The samples are nationally and sub-nationally representative. The data are open-source and can be used for policymaking, program planning, and research. PMA employs a multistage, stratified cluster sampling method, selecting households from designated clusters known as enumeration areas (EAs). In each EA, 35 households are randomly selected for interviews. For this analysis, we included data collected from eligible women based on responses to the household and female questionnaires. For more information, visit https://www.pmadata.org (25).

### 2.2 Study design, setting, and period

The Performance Monitoring for Action (PMA) project employed a longitudinal survey design to collect data in each country; it also included a cross-sectional sample. For this study, we used only the cross-sectional sample. Four SSA countries—Burkina Faso, Ethiopia, Kenya, and Uganda—were included in the analysis after applying exclusion criteria, using data from the most recent phase (2022–2024) available on the PMA website.

### 2.3 Sampling procedure, population, and sample size

**Figure 1:**
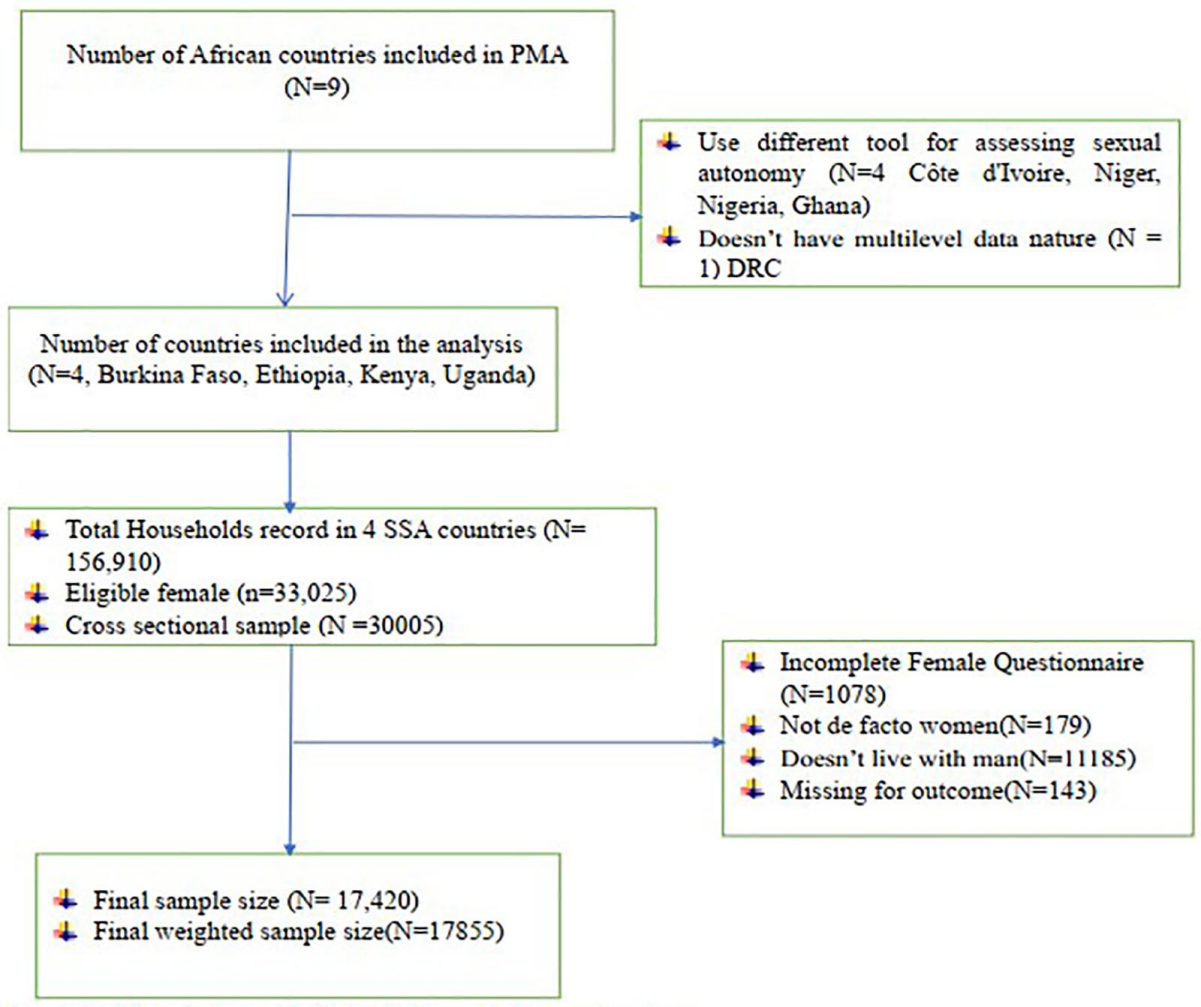
Flow chart to select the study population and country.

The source population comprises all women of reproductive age in Sub-Saharan Africa, while the study population includes women of reproductive age residing in four Sub-Saharan African countries: Burkina Faso, Ethiopia, Kenya, and Uganda. For this analysis, we included data collected from eligible women based on responses from household and female questionnaires. We excluded women who were single, divorced, or widowed, as well as those who were not de facto members of the household (i.e., women who did not sleep in the household the night before the survey). Finally, a total weighted sample of 17,855 de facto women of reproductive age was included in the analysis (Figure 1).

### 2.4 Variables of the study

The dependent variable in this study was sexual autonomy, defined as the presence of choice, and measured using a subscale from the Women's and Girls' Empowerment in Sexual and Reproductive Health (WGE-SRH) Index.

Some of the independent variables include socio-demographic factors such as age, place of residence, husband's education, and the respondent's education. Reproductive health variables encompass reproductive coercion, the presence of other wives, age at first marriage, and current use of family planning.

### 2.5 Measurement

The WGE-SRH Index differentiates between the existence of choice (sexual autonomy) and the exercise of that choice. It emphasizes that autonomy, self-efficacy, negotiation, and decision-making—although often combined into single indicators—should be treated as distinct constructs, as each independently influences sexual and reproductive health behaviors (4).

Therefore, this study relies on sexual autonomy, or the existence of choice. This sub-scale comprises four Likert-scale questions that assess women's perceived ability to make autonomous decisions regarding sexual activity with their partners. These items include: “If I refuse sex with my husband/partner, he may physically hurt me”, “If I refuse sex with my husband/partner, he may force me to have sex”, “If I show my husband/partner that I want to have sex, he may consider me promiscuous”, and “If I refuse sex with my husband/partner, he may stop supporting me”.

Each item used a five-point Likert scale, with 1 denoting “strongly disagree” and 5 denoting “strongly agree” to score each item. Higher sexual autonomy is indicated by lower total scores, which range from 4 to 20. According to psychometric assessments, the items demonstrated adequate internal consistency (Cronbach's alpha = 0.73) and constituted a single latent construct (eigenvalue = 1.65, with factor loadings greater than 0.5).

### 2.6 Operational definition

Sexual Autonomy: The total score was dichotomized using the median value (8) as the cutoff. Scores equal to or below the median were classified as high sexual autonomy (coded as 0), while scores above the median were classified as low sexual autonomy (coded as 1).

Media access was determined by combining access to TV and radio. If a respondent reported having access to either TV or radio, they were considered to have media access.

### 2.7 Data management and analysis

This study included data from four Sub-Saharan African countries. Prior to analysis, the data were weighted using the “svyset” command in STATA to account for the complex survey design and ensure representativeness by incorporating sampling weights, primary sampling units (EAs), and strata. Data cleaning, coding, and statistical analyses were performed using STATA version 17. Descriptive statistics, including proportions and frequencies, were presented through graphs, tables, and text.

Socioeconomic inequality was assessed using both the concentration curve and the concentration index. The concentration curve plots the cumulative proportion of the outcome variable (sexual autonomy) on the *y*-axis against the cumulative proportion of the population ranked by wealth, from the poorest to the richest, on the *x*-axis (26). Wealth quintiles were determined by assigning each household a score based on the number of assets owned, then dividing households into five groups: lowest, lower, middle, higher, and highest (27).

If the distribution of the outcome is equal across all wealth groups, the curve aligns with a 45-degree diagonal, known as the line of equality. A curve that lies above this line indicates that sexual autonomy is more concentrated among the pro-poor population, while a curve below the line suggests it is more common among the pro-rich.

To quantify and compare the extent of inequality in sexual autonomy, a binary outcome coded as 0 and 1, we applied the Erreygers Normalized Concentration Index (ECI), a modified version of the standard concentration index for bounded health variables (26, 28, 29).

To examine the relative contribution of different factors to the observed socioeconomic inequalities in sexual autonomy, we have applied the wag staff decomposition analysis.

The wag staff decomposition analysis has generated the coefficient, elasticity, concentration index, and the percent contribution. Elasticity quantifies the sensitivity to change of low sexual autonomy for each explanatory variable. Its value may be positive or negative, signifying a rise or fall in the inequality. The concentration index, displays the distribution of the variables according to wealth quintile. If the variable has a negative value, it is more concentrated among the poor households, and if it has a positive value, it is more concentrated among the richer households.

Regarding the percent contribution, a positive contribution indicates that the combined effect of a determinant's marginal effect (elasticity) and its distribution across wealth quintiles (concentration index) serves to increase inequality. This occurs when the determinant is either more common among poorer individuals and associated with a higher likelihood of low sexual autonomy, or more prevalent among wealthier individuals and associated with a lower likelihood of the outcome (30).

### 2.8 Ethical consideration

We conducted a secondary data analysis by extracting publicly available datasets from the PMA website, an open-source platform accessible to public health planners and researchers. All analyses were performed using anonymized data that is publicly available. The ethical clearance for each survey was obtained by the respective national institutions responsible for conducting the surveys. Additionally, the study was reviewed and approved by the Johns Hopkins University Bloomberg School of Public Health (JHSPH) Institutional Review Board (FWA00000287).

## 3 Results

### 3.1 Background characteristics of the respondents

According to the study, 39.69% of participants were between the ages of 25 and 34. In terms of education, 40.46% attended primary school. Among the husbands, 39.5% had also attended primary school. Additionally, 74.77% of the women lived in rural areas, and 21.85% resided in poor households. Furthermore, 85.9% of participants had access to media. More than a quarter of the participants (31.81%) were from Ethiopia (Table 1).

**Table 1:** Background characteristics of the study participants in four SSA countries, 2022–2024.

Variables	Categories	Sexual autonomy		Total weighted frequency
		High (*N* = 10,236) 57.33%	Low (*N* = 7,619) 42.67%	
Maternal age	15–24	2,077 (55.69)	1,653 (44.31)	3,730 (20.89)
	25–34	4,245 (59.92)	2,840 (40.08)	7,086 (39.69)
	35–49	3914 (55.6)	3125 (44.4)	7,039 (39.42)
Education	No	2,698 (48.67)	2845 (51.33)	5,542 (31.04)
	Primary	4038 (55.89)	3187 (44.11)	7,225 (40.46)
	Secondary	2501 (65.73)	1,304 (34.27)	3,806 (21.31)
	Higher	1000 (77.97)	283 (22.03)	1,283 (7.18)
Residence	Urban	3060 (67.92)	1445 (32.08)	4,505 (25.23)
	Rural	7177 (53.76)	6173 (46.24)	13,350 (74.77)
Wealth Index	Poorest	1835 (49.23)	1892 (50.77)	3,727 (20.87)
	Poor	2152 (55.4)	1732 (44.6)	3,884 (21.75)
	Medium	1908 (52.97)	1694 (47.03)	3,602 (20.18)
	Rich	1,980 (59.79)	1,694 (47.03)	3,312 (18.55)
	Richest	2,361 (70.92)	968 (29.08)	3,330 (18.65)
Husband education	No	2,497 (49.33)	2,566 (50.67)	5,063 (28.38)
	Primary	3,837 (54.40)	3,216 (45.60)	7,052 (39.53)
	secondary	2,542 (64.64)	1,391 (35.36)	3,933 (22.04)
	Higher	1,355 (75.61)	437 (24.39)	1,792 (10.05)
Media access	No	777 (46.70)	887 (53.30)	1,664 (9.32)
	Yes	9,459 (58.42)	6,732 (41.58)	16,191 (90.68)
Country	Burkina Faso	2,466 (54.27)	2,077 (45.73)	4,543 (25.44)
	Ethiopia	3,305 (58.18)	2,376 (41.82)	5,681 (31.81)
	Kenya	3,388 (65.90)	1,753 (34.10)	5,141 (28.79)
	Uganda	1,078 (43.29)	1,412 (56.71)	2,491 (13.95)

### 3.2 Reproductive health-related characteristics of the respondents in four Sub-Saharan African countries, 2022–2024

Among the participants, 14.36% reported living with a man without being legally married. More than half (54.14%) had never used any form of contraception, whether modern or traditional. Additionally, 9.38% reported experiencing pregnancy coercion, and 20.7% stated that their husbands had other wives. Nearly half of the participants (46.10%) also reported that their first sexual experience occurred between the ages of 15 and 17 (Table 2).

**Table 2:** Reproductive health related characteristics of the respondents in four Sub-Saharan Africa countries, 2022–2024.

Variables	Categories	Sexual autonomy		Total weighted frequency
		High (*N* = 10,236) 57.33%	Low (*N* = 7,619) 42.67%	
Marital type	Married	8,943 (58.48)	6,348 (41.52)	15,291 (85.64)
	Cohabiting	1,294 (50.46)	1,270 (49.54)	2,564 (14.36)
contraceptive use	No	5,248 (54.28)	4,419 (45.72)	9,667 (54.14)
	Yes	4,989 (60.93)	3,199 (39.07)	8,188 (45.86)
Pregnancy coercion	No	9,693 (59.91)	6,487 (40.09)	16,180 (90.62)
	Yes	543 (32.43)	1,132 (67.57)	1,675 (9.38)
Other wife	No	8,391 (59.26)	5,768 (40.74)	14,159 (79.30)
	Yes	1,845 (49.92)	1,851 (50.08)	3,696 (20.70)
Age at first sex	<15	1,082 (51.20)	1,031 (48.80)	2,114 (11.84)
	15–17	4,560 (55.40)	3,671944.60)	8,231 (46.10)
	≥18	4,594 (61.17)	2,917 (38.83)	7,511 (42.06)

### 3.3 Low sexual autonomy across explanatory variables

**Figure 2:**
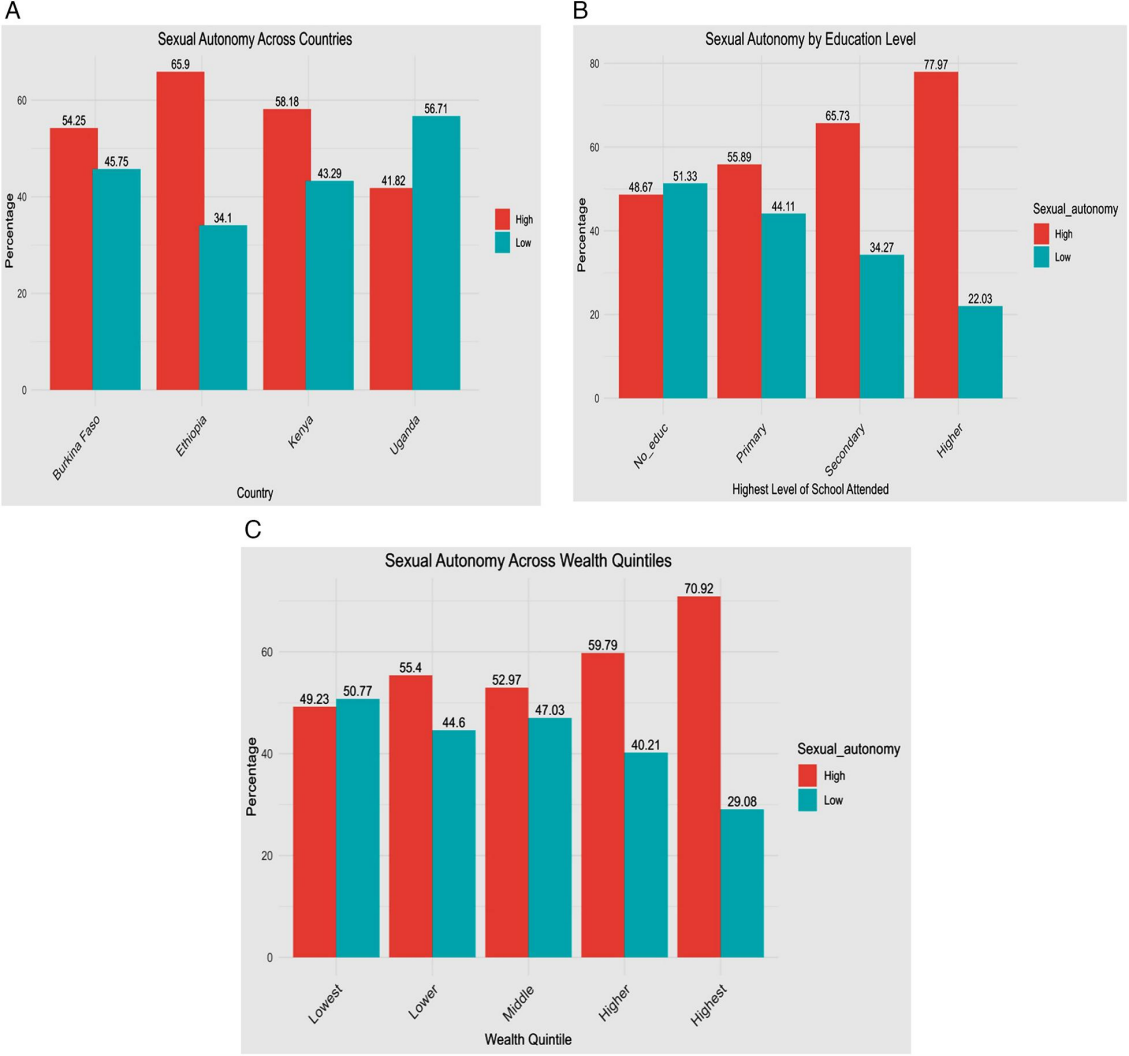
Sexual autonomy among reproductive-age women in four SSA countries, 2022–2024: **(A)** across countries (Burkina Faso, Ethiopia, Kenya, and Uganda); **(B)** by education level; and **(C)** across wealth quintiles.

The bar chart shows that Uganda has a higher proportion of women with low sexual autonomy (56.51%) compared to the other country, while Ethiopia has a lower proportion (34.1%) (Figure 2A). The proportion of women with low sexual autonomy decreases from 48.67% among those with no education to 22.03% among those with higher education (Figure 2B). A similar trend is observed across wealth quintiles, with the proportion declining from 49.23% in the lowest quintile to 22.08% in the highest quintile (Figure 2C).

**Figure 3:**
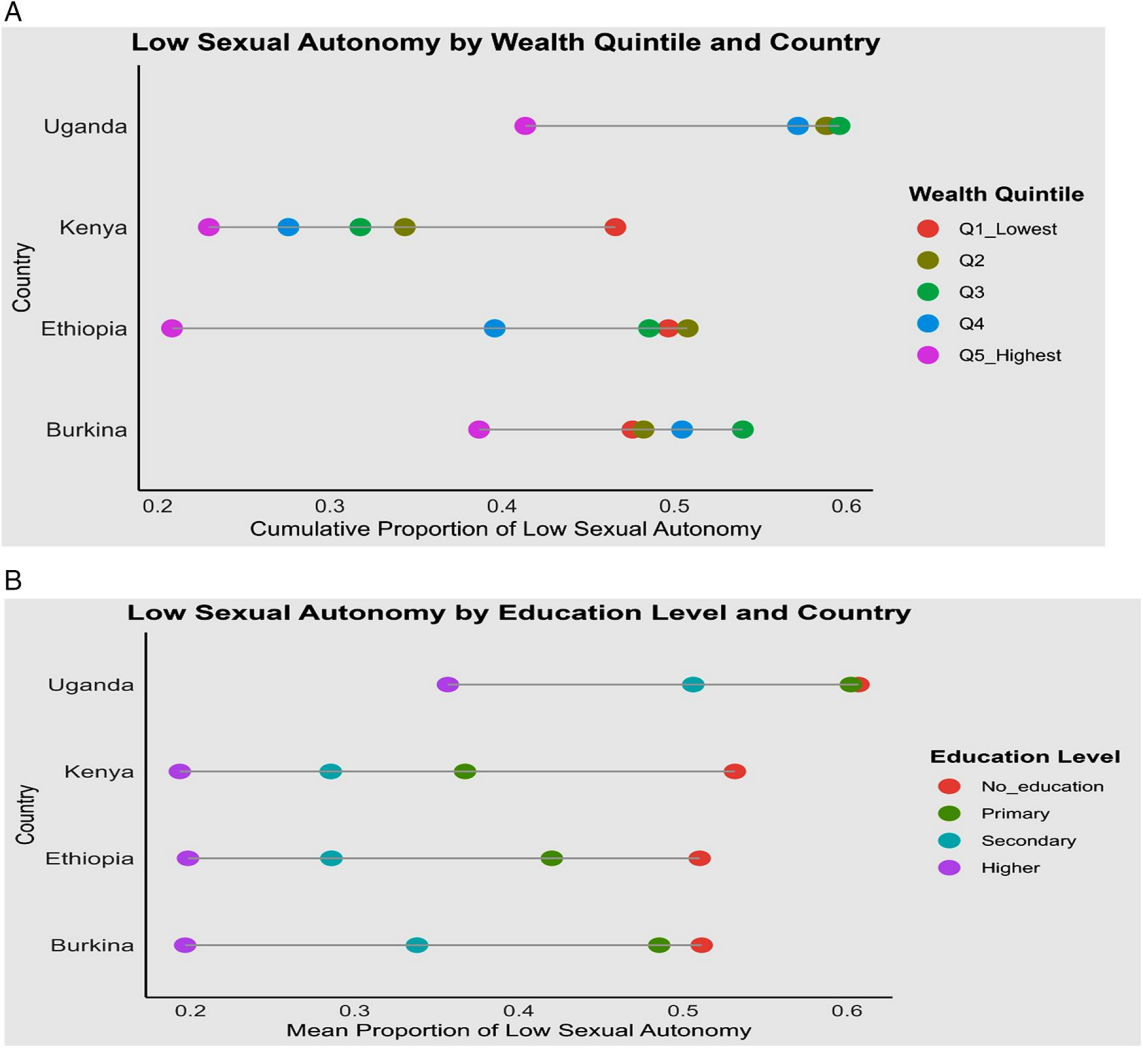
Equiplot showing proportion of low sexual autonomy among reproductive-age women in four SSA countries, 2022–2024 across wealth quintile **(A)** and educational level **(B)**.

From the equiplot, we observe that the proportion of low sexual autonomy increases as we move from the highest wealth quintile to the lowest. In Ethiopia and Uganda, the gap between the highest (Q5) and the fourth (Q4) wealth quintiles is substantial, indicating that wealth significantly influences sexual autonomy status. In contrast, Kenya and Burkina Faso show minimal differences between these quintiles (Figure 3A). Regarding education levels, a large gap in the proportion of low sexual autonomy exists between individuals with higher education and those with secondary education across the four countries. However, in Uganda and Burkina Faso, the baseline level of low sexual autonomy remains high regardless of wealth quintile (Figure 3B).

### 3.4 Pooled prevalence of low sexual autonomy among reproductive-age women in four SSA countries, 2022–2024

**Figure 4:**
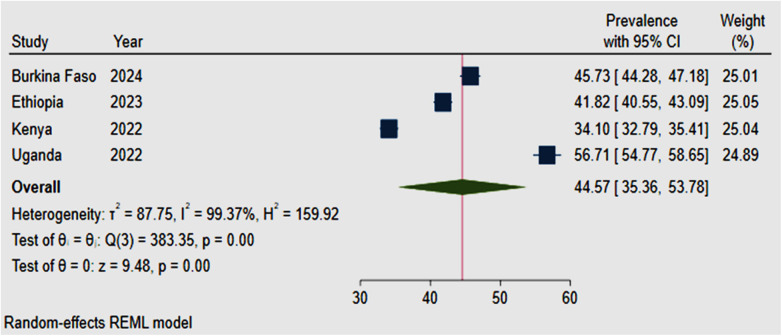
Forest plot for the pooled prevalence of low sexual autonomy among reproductive-age women in four SSA countries, 2022–2024.

The pooled prevalence of low sexual autonomy in four SSA countries was found to be 44.57% (95% CI: 33.36, 53.78) with significant heterogeneity (I^2^ = 99.37%, *p* < 0.001). The higher prevalence was observed in Uganda, 56.71% (95% CI: 54.77, 58.65), while the lower prevalence was observed in Kenya, 34.10% (95% CI: 32.79, 35.41) (Figure 4).

### 3.5 Socio-economic inequality of low sexual autonomy among reproductive-age women in four SSA countries 2022–2024

**Figure 5:**
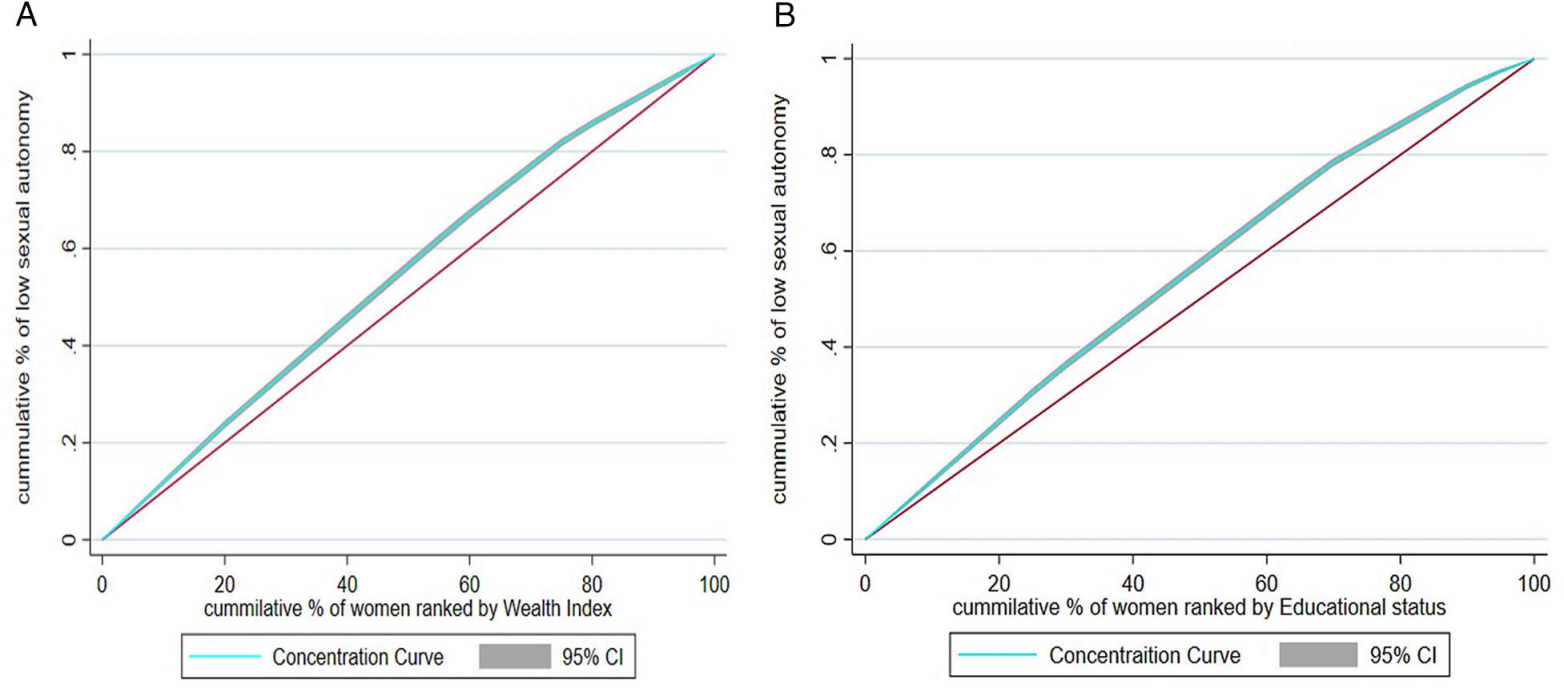
Wealth related inequality **(A)** and education related inequality **(B)** of low sexual autonomy among reproductive-age women in four SSA countries, 2022–2024.

The weighted Erreygers normalized concentration index for low sexual autonomy was −0.184 with a standard error of 0.021 (*P* < 0.0001). This indicated that low sexual autonomy was significantly concentrated among individuals from lower-income households. Likewise, the corresponding concentration curve (Figure 5A) lay above the line of equality, showing that low sexual autonomy was predominantly distributed among the poor.

Similarly, the weighted Erreygers normalized concentration index for low sexual autonomy with respect to educational status was −0.212, with a standard error of 0.02 (*P* < 0.0001). This also revealed a significant concentration of low sexual autonomy among less educated women. The concentration curve shown in Figure 5B supported this, as it appeared above the line of equality, indicating a higher concentration of low sexual autonomy among the less-educated group.

### 3.6 Decomposing the socio-economic inequality in low sexual autonomy among reproductive-age women in four SSA countries, 2022–2024

A decomposition analysis was conducted to examine the contribution of each independent variable to socio-economic inequality. The coefficients, elasticity's, concentration indices, and percentage contributions of each variable were presented in tables.

A decomposition analysis was conducted to examine the contribution of each independent variable to socioeconomic inequality. The coefficients, elasticities, concentration indices, and percentage contributions of each variable were presented in tables.

Women aged 15–24, husband's education (primary, secondary, or higher), contraceptive use, women's education (primary, secondary, or higher), media access, and all wealth indexes except the middle category have negative values. In contrast, age 25–34, rural residence, first sexual intercourse before age 15, pregnancy coercion, and the presence of other wives have positive signs.

For instance, the elasticity value for rural residence (0.1315) indicates that if women shifted from urban to rural residence, the proportion of low sexual autonomy would increase by approximately 13.15%. Similarly, the elasticity for media access (–0.1527) suggests that if women shifted from no media exposure to exposure (radio, TV, or mobile), the proportion of low sexual autonomy inequality would decrease by about 15.27%.

This study found that maternal age between 25 and 34, women and their husbands with secondary or higher education, contraceptive use, media access, and first sexual activity before age 15 are more concentrated among wealthier households. Conversely, maternal age between 15 and 24, women and their husbands with only primary education, urban residence, pregnancy coercion, and the presence of other wives have a negative value, meaning these variables are more concentrated in the lower wealth quintiles.

From the percent contribution, both positive and negative contributions were observed. The factors included in this decomposition explained 90.28% of the observed inequality, with the remaining 9.72% likely attributable to residual variables. Residence was the largest contributor to overall socio-economic inequality, accounting for 38.62%. This indicates that rural residence is disproportionately concentrated among poorer households (CI = −0.539) and is positively associated with low sexual autonomy (E = 0.135). Other significant contributors included media access (16.52%), wealth quintile (13.71%), women's education (8.87%), and husband's education (7.24%). Although wealth status alone explained 13.71% of the inequality, the majority was attributed to residence and media access (Table 3).

**Table 3:** Contributing factors for the socio-economic inequality of low sexual autonomy among reproductive age women in 4 African countries, 2022–2024.

Variables	Category	Coefficient	Elasticity	Concentration index	Absolute contribution	% Contribution
Age	15–24*	0.0208	0.0174	−0.0217	−0.0004	0.21
	25–34*	−0.0136	−0.0215	0.0756	−0.0016	0.89
	35–49	Ref			−0.0020	
Subtotal						1.11
Husband Education	No	Ref				
	Primary	−0.0106	−0.0167	−0.1499	0.0025	−1.37
	Secondary*	−0.0537	−0.0473	0.1832	−0.0087	4.72
	Higher*	−0.0916	−0.0368	0.2091	−0.0077	4.19
Sub total					−0.0139	7.24
Residence	Urban	Ref				
	Rural*	0.044	0.1315	−0.539	−0.0709	38.62
Contraceptive use	No	Ref				
	Yes*	−0.0073	−0.0134	0.1414	−0.0019	1.03
Women educational level	No	Ref				
	Primary*	−0.041	−0.0648	−0.127	−0.0083	−4.48
	Secondary*	−0.088	−0.075	0.237	−0.017	9.68
	Higher*	−0.149	−0.043	0.1568	−0.0067	3.67
Subtotal					−0.032	8.87
Media access	No	Ref				
	Yes*	0.0421	−0.1527	0.1984	−0.030	16.5
Age at first sex	<15*	0.0227	0.0107	0.0756	−0.0016	0.46
	15–17	−0.0051	−0.0094	−0.1406	0.0013	−0.72
	≥18	Ref			−0.0003	
Sub total						−0.26
Wealth index	Poorest	Ref				
	Poorer*	−0.0341	−0.0296	−0.3176	0.0094	−5.13
	Middle*	0.0029	0.0023	0.0438	0.0001	−0.06
	Richer	−0.0236	−0.0175	0.3276	−0.0057	3.12
	Richest	−0.064	−0.0477	0.6068	−0.0290	15.78
Subtotal					−0.0252	13.71
Pregnancy coercion	No	Ref				
	Yes	0.2549*	0.0956	−0.0361	−0.0034	1.88
Other wives	No	Ref				
	Yes	0.0390*	0.322	−0.0897	−0.0029	1.58

* *p* < 0.05.

## 4 Discussion

The findings of this study revealed a significant level of socioeconomic inequality in low sexual autonomy across four Sub-Saharan African countries. Low sexual autonomy is disproportionately concentrated among women in the lowest wealth quintiles and those with lower levels of education. The decomposition analysis showed that 13.1% of this inequality was attributable to the direct effect of wealth status. Additionally, the equiplot analysis highlights a gap in the proportion of low sexual autonomy between the wealthiest and poorest quintiles, particularly in Ethiopia, Uganda, and Burkina Faso, indicating that wealth status significantly influences sexual autonomy. The study also underscores the association between socioeconomic factors, such as wealth, and sexual autonomy (18, 20, 31–34). This may be because women from poorer households tend to be more financially and socially dependent on their husbands, which can limit their confidence and ability to freely express their sexual preferences and negotiate their rights (35).

The pooled prevalence of low sexual autonomy was 44.57%. This finding aligns with the UNFPA report, which reported a prevalence of 45% (11). However, it is higher than rates reported in other studies from Sub-Saharan Africa, which found prevalence of 27% and 16.65% (14, 15). These discrepancies may be attributed to differences in measurement tools; for example, some studies used a simple yes/no question, where either response was considered indicative of sexual autonomy, whereas our study employed a Likert scale, allowing for more nuanced responses. Among the countries studied, Ethiopia exhibited a comparatively lower proportion of low sexual autonomy, which may be due to the implementation of programs focused on improving sexual and reproductive health rights (SRHR) (36).

From the decomposition analysis, the primary contributor to overall socioeconomic inequality was rural residence, accounting for 38.62%. This indicates that living in rural areas plays a significant role in the disparity in sexual autonomy between the poor and the wealthy. This is because rural residence is more common among poorer populations and is directly associated with lower sexual autonomy. The impact of rural residence on reduced sexual autonomy is also supported by various other studies (20, 34, 37). This may be attributed to limited access to healthcare services in rural areas and the strong influence of traditional societal norms, which can restrict women's ability to freely express their needs and rights. Compared to urban women, rural women are more likely to engage in sexual activity out of fear or to satisfy their husbands’ desires, rather than based on their own will or pleasure (38, 39).

In addition to urban residence, media exposure (16.52%) also significantly contributes to inequality in low sexual autonomy. This is because media exposure is concentrated among wealthier individuals and is associated with a lower likelihood of experiencing low sexual autonomy. Evidence from sub-Saharan Africa highlights the positive impact of media access on sexual negotiation and autonomy (14, 40). A possible explanation is that media exposure may enhance women's knowledge of sexual and reproductive health, thereby improving their decision-making abilities (41).

Education also contributes to inequality, accounting for 8.87%. Specifically, secondary and higher education were concentrated among wealthier individuals and inversely associated with low sexual autonomy. This finding is supported by a previous study that revealed a correlation between education and sexual autonomy (31, 32). The equiplot further revealed a wider gap in sexual autonomy between women with no education and those with higher education across all four countries. Education may enhance women's ability to make informed choices by increasing their awareness of rights and available services. It may also reduce their vulnerability to traditional societal and gender norms that often restrict women's agency (42).

Another contributor to overall socioeconomic inequality was the husband's educational status. This finding is supported by previous studies conducted in Sub-Saharan Africa and Ghana, which demonstrated that a husband's education influences the refusal of risky sex (31, 32, 43). This may be because an educated husband is more likely to respect his wife's choices and allow her to exercise her rights. Additionally, he may be more open to discussion (44).

Given the large sample size and its comprehensive representation at both national and subnational levels, the findings are generally considered broadly applicable. However, the study has certain limitations. There may be social desirability bias influencing participants' responses. Additionally, some variables, such as religion, were excluded due to their unavailability in the Uganda dataset.

## 5 Conclusion

This study identified significant socioeconomic inequalities in sexual autonomy across the four SSA countries (Burkina Faso, Ethiopia, Kenya, and Uganda). According to the decomposition analysis, rural residence was the most significant contributor to overall socioeconomic inequality, followed by access to media, economic status, women's educational attainment, and their husbands' education levels. Policymakers and public health planners focused on gender equality and reproductive health must design and implement targeted interventions that support women from low-income households, with lower levels of education, or living in rural areas. These efforts will help promote women's empowerment and advance gender equality.

## Data Availability

The original contributions presented in the study are included in the article/Supplementary Material, further inquiries can be directed to the corresponding author.
